# Potential Role of CCN Proteins in Breast Cancer: Therapeutic Advances and Perspectives

**DOI:** 10.3390/curroncol28060417

**Published:** 2021-11-26

**Authors:** Kazi Ahsan Ahmed, Tasnin Al Hasib, Shamrat Kumar Paul, Md. Saddam, Afsana Mimi, Abu Saim Mohammad Saikat, Hasan Al Faruque, Md. Ataur Rahman, Md. Jamal Uddin, Bonglee Kim

**Affiliations:** 1 ABEx Bio-Research Center, East Azampur, Dhaka 1230, Bangladesh; kaziahsanahmed.bmb@gmail.com (K.A.A.); hasib0608@gmail.com (T.A.H.); shamratpaul.bmb@gmail.com (S.K.P.); hasan_vt@yahoo.com (H.A.F.); 2 Department of Biochemistry and Molecular Biology, Life Science Faculty, Bangabandhu Sheikh Mujibur Rahman Science and Technology University, Gopalganj 8100, Bangladesh; saddubmb@gmail.com (M.S.); afsanamimijbmb@gmail.com (A.M.); asmsaikat.bmb@gmail.com (A.S.M.S.); 3 Bio-Science Research Initiative, Gopalganj 8100, Bangladesh; 4 Companion Diagnostics and Medical Technology Research Group, Daegu Gyeongbuk Institute of Science and Technology (DGIST), Daegu 42988, Korea; 5 Department of Pathology, College of Korean Medicine, Kyung Hee University, Seoul 02447, Korea; 6 Graduate School of Pharmaceutical Sciences, College of Pharmacy, Ewha Women’s University, Seoul 03760, Korea

**Keywords:** breast cancer, CCN proteins, tumorigenesis, therapeutic advances, prospects

## Abstract

CCNs are a specific type of matricellular protein, which are essential signaling molecules, and play multiple roles in multicellular eukaryotes. This family of proteins consists of six separate members, which exist only in vertebrates. The architecture of CCN proteins is multi-modular comprising four distinct modules. CCN Proteins achieve their primary functional activities by binding with several integrin7 receptors. The CCN family has been linked to cell adhesion, chemotaxis and migration, mitogenesis, cell survival, angiogenesis, differentiation, tumorigenesis, chondrogenesis, and wound healing, among other biological interactions. Breast cancer is the most commonly diagnosed cancer worldwide and CCN regulated breast cancer stands at the top. A favorable or unfavorable association between various CCNs has been reported in patients with breast carcinomas. The pro-tumorigenic CCN1, CCN2, CCN3, and CCN4 may lead to human breast cancer, although the anti-tumorigenic actions of CCN5 and CCN6 are also present. Several studies have been conducted on CCN proteins and cancer in recent years. CCN1 and CCN3 have been shown to exhibit a dual nature of tumor inhibition and tumor suppression to some extent in quiet recent time. Pharmacological advances in treating breast cancer by targeting CCN proteins are also reported. In our study, we intend to provide an overview of these research works while keeping breast cancer in focus. This information may facilitate early diagnosis, early prognosis and the development of new therapeutic strategies.

## 1. Introduction

In 2020 alone, almost 2.3 million women were diagnosed with breast cancer worldwide. Breast cancer is the most frequently reported cancer and the leading cause of cancer-related mortality in women globally [1,2]. Members of the CCN family are matricellular proteins that are present in the extracellular matrix (ECM). Unlike most other ECM proteins, they serve a regulatory role instead of a structural [3]. The CCN family comprises six homologous proteins (CCN1–CCN6). This protein family are multi modular proteins having four functional modules, but CCN5 lacks the C-terminal (CT) module [4]. The human breast is composed of lobes that produce milk and ducts that carry the milk to the nipple, and all of these are surrounded by muscle and fat (adipose) tissues [5].

Since CCN proteins have diverse functionality, their involvement in cancer and breast cancer in particular, lacks uniformity. Increased expression of CCN1, CCN2, CCN3 and CCN4 is closely related with cell adhesion, proliferation and migration, and thus they can contribute directly to tumorigenesis, tumor development and metastases [3,6,7,8,9,10]. Overexpression of CCN3 is associated with breast cancer metastasis to distant organs [11]. CCN1, CCN3, CCN5 and CCN6 may play dual roles; their expression can inhibit breast cancer growth, and their absence can induce carcinoma [12,13,14,15]. In breast carcinoma, the positive or negative relationship of CCNs (CCN1–CCN6) has been specified in this review. The association of CCN proteins with numerous activities indicates that any alterations or malfunction of CCN protein activity can trigger a cascade of physiological events in humans. This article will help in the design of drugs and therapeutics targeting CCN proteins in breast cancer.

## 2. Methods

In this project, we retrieved the literature from online public databases such as PubMed and Google Scholar using keywords, including CCN protein, breast cancer, metastasis, mechanisms, therapeutic advances, and prospects, and cite each resource with references. All the graphical works are processed through the Inkscape software package [16,17,18].

## 3. Nomenclature

The six matricellular proteins (CCN1–CCN6) belong to the CCN protein family. Several genes encoding the CCNs have been documented to be composed of four motives (cysteine-rich) with a good multi-modular structure (conserved). It is estimated that the N-terminal motif bears the consensus sequence (GCGCCXXC), which has a highly conserved IGF-binding sequence [19]. The CCN family includes CCN1/CYR61 (cystein-rich 61], CCN2/CTGF (connective tissue growth factor), CCN3/NOV (nephroblastoma overexpressed), and also CCN4/WISP-1, CCN5/WISP-2, CCN6/WISP-3 (Wnt-inducible secreted proteins) [10,20]. The multi-modular organization implies that CCNs can act mutually with other proteins to exercise similar functions. CCN’s biological functions may be dependent on the availability of interacting substrates and proteins. This would also describe the peculiar biochemical consequences and the wide spectrum of pathological and physiological roles of the members of the CCN family. The CCN protein family is known to mediate epithelial–stromal crosstalk. Therefore, they interact with some key signaling molecules especially cell surface integrins including (α_2_β_1_, α_5_β_1_, α_6_β_1_, α_v_β_5_, α_IIb_β_3_, α_M_β_2_, and α_D_β_2_) [21,22]. Human Genome Organization (HUGO) nomenclature committee renamed the CCN proteins as cellular communication network factors [23].

## 4. CCN Gene Family

The six different members of the CCN gene family includes Cyr61 (CCN1), CTGF (CCN2), NOV (CCN3), WISP-1 (CCN4), WISP-2 (CCN5), and WISP-3 (CCN6) [24]. Biological functions, such as cell growth, ECM regulation, cell adhesion, migration, wound healing and angiogenesis, and diseases including fibrosis, cancer and vascular diseases are mostly regulated by the expression of the CCN gene family. CCN proteins are not growth factors; but, they can modify the signaling of other molecules associated with the ECM [20]. Cyr61 (CCN1) is an integrin receptor and an angiogenesis inducer in breast cancer [25]. CTGF (CCN2, connective tissue growth factor) was initially recognized as a protein secreted by human endothelial cultured cells. The protein encoded by the CCN2/CTGF gene is a mitogen associated with platelet-derived growth factors. CCN4 (WNT1) is a matricellular protein with a cysteine-rich motif. The WNT1 gene is involved in tumor development through the activation of β-catenin, and is considered to be a novel target against toxic cell degeneration. WNT1 is activated in the Wnt/β-catenin signaling pathway, as a regulator of cell functions [25].

CCN5, previously known as WISP-2, rCop-1, COP-1, HICP and CTGF-L, can perform a regulatory role by reducing epithelial-to-mesenchymal transition (EMT) in both breast and pancreatic cancer cells. In noninvasive breast cancer cell lines, several genetic exposures disrupt CCN5, which is responsible for invasive cancer phenotypes. It was detected as a heparin-induced gene and acted as a growth-arresting gene in vascular smooth muscle cells [26]. CCN6/WISP3 is rich in (IGF)binding protein domain. Additionally, the WISP3 gene plays an important role in the mitochondrial electron transport system [14].

## 5. Molecular Makeup of CCN Proteins

The molecular makeup of CCNs is conserved among members and various species. CCNs have four exclusive modules with noteworthy structural characteristics and minor exceptions [27]. The IGF-IGFBP (insulin-like growth factor binding protein-like), von Willebrand factor type C (VWC), thrombospondin 1 (TSP1), and cysteine-knot-containing (CT) are named Modules I, II, III, and IV, respectively. [28]. CCN5 is the only exception to the alignment of these modules in the CCN family, as it lacks the fourth CT module. The CCN’s cysteine ratio is comparatively larger, about 10% by mass, containing 38 cysteines separated into 17 retained zones, and extended across four fields. Some dissimilarities have been observed between CCN5 (WISP-2) and CCN6 (WISP-3). CCN5 lacks 10 cysteine amino acids as it has no CT domain [29]. The two N-terminal Modules (I and II) and two C-terminal Modules (III and IV) of the CCN proteins are separated by a linker with a variable amino acid composition. Modules II and III are connected by the “hinge region”, which is sensitive to cleavage by proteinases. A conserved exon encodes these structural modules, and the CCN genes are speculated to be products of exon shuffling [22,30].

The types of binding partners and ligands depend on several molecular structures of the functional domains of the CCN protein. The binding partners are specific to domains: IGFs attach to IGFBP; BMPs, TGF-β, and integrins interact with VWC; VEGF, HSPGs, LDL receptor proteins (LRPs), and integrins interact with TSP-1, and VEGF, integrins, LRPs, Notch1, fibulin-1C, HSPGs, and integrins interact with CT [10,31]. Thus, their action, half-life, bioavailability, binding to other protein moieties, and regulation time of the CCN portion are directly associated with the modular configuration.

## 6. Potential Role of CCN Proteins in Tumorigenesis and Breast Cancer

CCN proteins are involved in different aspects of cancer development. Individual CCN proteins may also act differently in many cancer types via various signaling pathways, even though they share structural homology [32]. Some of them have already been found to induce cancer; some are found to be involved in malignancy. CCN proteins exert their functions in two different ways: interaction with cell surface receptors and with receptor ligands. Studies of CCN proteins at the molecular level show that extracellular signal-regulated kinases (ERKs), phosphoinositide 3-kinase (PI3K), and small GTPases of the Rho family act at the activation site to trigger the signaling cascades in the cellular context [20,21,32]. CCN proteins can modify the signals of several proteins, including integrins, Wnt, and transforming growth factor-β (TGF-β). These proteins are correlated with cellular proliferation and studies indicate their association with several cancer risk factors. Chronic inflammation is a major risk factor [10,33] (see Table 1).

**Figure 1:**
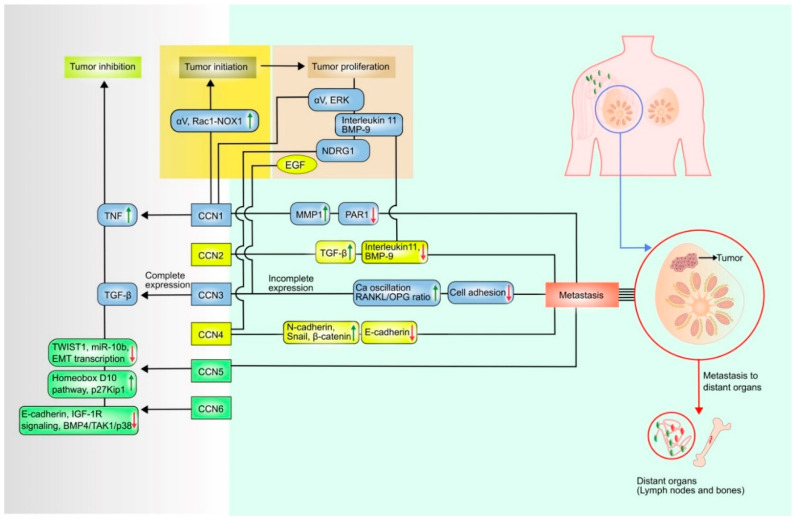
Dual role of CCN proteins in breast cancer. This schematic diagram illustrates that CCN1 initiate breast tumor by modulating αV integrin proteins and Rac1-NOX complex formation. CCN1 proliferate the tumor via αV and extracellular-signal-regulated kinase (ERK), CCN2 via interleukin11, bone morphogenetic protein-9 (BMP-9), CCN3 via epidermal growth factor (EGF) and CCN4 via N-Myc downstream regulated 1 (NDRG1). CCN1, CCN2, CCN3, CCN4 can upregulate matrix metalloproteinase-1 (MMP-1), transforming growth factor beta (TGFβ), Ca oscillation and RANKL/OPG ratio, N-cadherin, snail and β-catenin proteins, respectively. They can also down-regulate protease-activated receptor (PAR), interleukin 11 and BMP-9, cell adhesion, E-cadherin respectively, and metastasize the tumor to distant organs. CCN1 can inhibit tumor growth by upregulating tumor necrosis factor (TNF), CCN3 by binding with TGFβ, CCN5 by upregulating homeobox D10 pathway, p27Kip1 and downregulating twist-related protein 1 (TWIST1), microRNA 10b (miR-10b), epithelial–mesenchymal transition transcription (EMT transcription), CCN6 by downregulating E-cadherin expression, IGF-1R (insulin-like growth factor type 1 receptor) signaling, BMP4 (bone morphogenetic protein 4)/TAK1(transforming growth factor-β-activated kinase 1)/p38.

Deregulated healing of lesions is also a risk factor in cancer progression. In the early stages of neoplastic transformation, inflammation is responsible for speeding up the development of primary neoplasia into cancer by inducing mutagens, such as reactive oxygen species [21]. Aberrant expression of some CCN proteins is associated with the regulation of diverse inflammatory mediators, including TGF-β, prostaglandins, and ECM enzymes. Moreover, estrogenic influence, pro-inflammatory adipokines such as tumor necrosis factor-alpha (TNFα), and leptin are also reported to be tumor-associated biochemical risk factors [44]. In breast tumors, both the steroid-dependent proteins CCN1 and CCN2 are overexpressed; these proteins are also estrogen-inducible [41]. Estrogenic GPR30 signaling promotes the proliferation and migration in breast cancer cells via CCN2 [41]. CCN1, CCN2, CCN3, and CCN4 are pro-tumorigenic and may be responsible for human breast carcinoma; while CCN1, CCN3, CCN5 and CCN6 have anti-tumorigenic effects (see Figure 1). CCN1, CCN3, CCN5, CCN6 play a critical role in survival in breast cancer.

### 6.1. CCN1: CYR61

Aberrant expression of CCN1, commonly known as cysteine-rich angiogenic inducer 61 (CRY61), has been found to be related to various classes and categories of cancer [45]. CCN1 proteins connect the extracellular matrix with cells via the cell surface integrin proteins. As the name CYR61 suggests, they promote angiogenesis that increases tumor growth, mainly by binding with αV β3 integrins, which stimulates the secretion of vascular endothelial growth factor (VEGF) and transforming growth factor β [35]. CCN1 proteins are found to be associated with tumor cell proliferation as well as migration and adhesion of tumor cells [6]. The elevated level of CCN1 is associated with lymph node metastases and a worse chance of recovery in breast cancer patients. Overexpression of CCN1 in breast cancer cells is found to be associated with increased tumor size and vascularization [46].

CCN1 mediate cellular adhesion by binding with integrin α-v β-1 and α-v β-5 [47]. In ER-positive breast cancer, CCN1 activates MMP1 which stimulates PAR1 to increase malignancy. Malignancy of cancer cells is a multistep process and involves a variety of signaling molecules, events and mediators. Both the migration and adhesion of cancer cells are crucial for tumor cell metastasis as malignancy of cancer cells involves dissemination and adhesion. CCN1 can increase tumor invasion and metastasis through various mediators. The migratory activity of the protein mainly depends on the C-terminal (CT) domain. Thus, the CT domain of the proteins can be a critical modulator of breast cancer metastasis [37]. Ectopic expression of CCN1 can stimulate the dissemination of cancer cells from the primary tumor site, and can facilitate their binding to other tissues by modulating integrin proteins [36].

Furthermore, the cysteine-rich conserved domains of CCN1 protein indicate that specific three dimensional conformation might be involved in achieving certain functionality [48]. CCN1 is currently known as one of the potential targets for chemotherapy against breast cancers [27]. The tumor inhibitory function of CCN1 has been revealed in quite recent times. CCN1 can mediate senescence and apoptosis through tumor necrosis factor α (TNFα). Thus, the protein can suppress tumor proliferation by prompting senescence and apoptosis [13]. Paradoxically, therapeutic measures have also been developed to inhibit breast cancer development targeting CCN1.

### 6.2. CCN2: CTGF

The first domain of CCN2 exhibit very low affinity with insulin-like growth factor (IGF). Thus, CCN2 achieves its functionality mainly through the three other domains [49]. These domains bind to transforming growth factor-β (TGF-β), bone morphogenetic proteins (BMPs) as well as αvβ3, αvβ5 integrins to regulate their functionality [38,50]. Increased expression of CCN2 leads to an increase of breast cancer metastasis, probably abusing TGF-β and BMP growth factors. CCN2 mediates osteolytic metastasis of breast cancer via the protein kinase A- and protein kinase C- dependent activation of ERK [3]. In ER-negative human breast cancer cells, induction of GPR30 signaling mediated by estrogen or hydroxytamoxifen (OHT) activates a cascade of transcription factor networks. The stimulation of GPR30 by OHT promotes CCN2 proteins in breast cancer associated fibroblasts (CAFs), which contributes to the invasive behavior of breast tumors. Increased levels of endogenous estrogen or administration of OHT for endocrine therapy equally act as promoting factors for CCN2 [51]. Bone Morphogenetic Proteins are growth factors that participate in the formation of bone and cartilage. In triple-negative breast cancer cells, CCN2 is found to increase metastasis to bone through downregulating BMP-9 [38]. A study conducted by Chien et al. reported that CCN2 expression in breast cancer cells results in increased migration, angiogenesis resulting in a worse prognosis. Furthermore, ectopic expression of CCN2 in breast cancer cell lines also stimulates angiogenesis and migration, where the CT domain of CCN2 protein plays a pivotal role [7].

### 6.3. CCN3: NOV

CCN3, previously known as NOV (nephroblastoma overexpressed), was one of the first identified members of the CCN protein family. The CCN3 gene may be transcriptionally upregulated by the tumor suppressor p53. The complete expression of the protein inhibits fibroblastic cells reflecting its tumor suppressing properties [52,53]. CCN3 inhibits cellular proliferation mainly by binding with TGF-β proteins. However, CCN3 expression at the cellular level can be unfinished. The impaired expression that only includes TSP-1 and CT domains can be involved in increasing transcriptional activity. Transcriptional upregulation is accompanied by higher expression of EGFr signaling. This course can rationalize the presence of impaired CCN3 proteins in the nucleus of malignant cancer cells [39]. “Complete” CCN3 (expression of all four of CCN3 domains) is an anti-proliferative protein but can be involved in cancer malignancy. CCN3 is found to be involved in cellular motility mediated by cadherin. It can interact with metastasis promoter S100A4 and modulate cytoskeletal dynamics and the proliferation of cancer cells [12]. Overexpression of the CCN protein may contribute to the aggressive behavior of osteolytic breast cancer by increasing nuclear translocation of nuclear factor of activated T cells c1 (NFATc1) and calcium oscillations [11].

### 6.4. CCN4: WISP-1

Limited evidence indicates that CCN4, commonly known as WISP-1, has pro-tumorigenic features and functionality in breast cancer. WISP-1 mRNA expression is relatively higher in breast cancer patients than in healthy patients. WISP-1 overexpression has been found to facilitate the initiation of MCF7 tumors in xenograft studies [9]. Quite the opposite picture has been revealed in the case of breast cancer development. A lower level of WISP-1 was found in tumor cells than in normal breast tissue which is far lower in more invasive tumor types. Evidence also showed that CCN4 represses E cadherin activity and facilitates N cadherin, snail and β catenin upregulation, consequently facilitating cancer metastasis. Thus, it can be concluded that WISP-1 is not crucial for tumorigenesis and can repress tumor growth in some carcinomas but participate in cancer progression and metastases [8,9]. CCN4 promotes tumorigenesis mainly by inhibiting N-myc downstream regulated 1 (NDRG), a breast tumor suppressor gene [9].

### 6.5. CCN5: WISP-2

CCN5 is the only protein in the CCN protein family where the CT module containing carboxyl domain is absent and it plays an anti-tumorigenic effect on breast cancer. CCN5 acts as a tumor suppressor in the Erα-positive cell line by binding to the promoter of TGF-β in the nucleus to suppress its expression [54]. CCN5 also suppresses EMT (epithelial–mesenchymal transition) expression which also induces epithelial cell migration by inhibiting EMT gene transcription. However, in case of more invasive breast cancer such as ER-negative breast cancer, CCN5 expression is minute [41]. Expression of CCN5 is predominant in preneoplastic disorders, such as atypical ductal hyperplasia and noninvasive ductal carcinoma in situ (DCIS). CCN5 inhibits TWIST1 expression which in turn suppresses miRNA-10b. miRNA-10b expression negatively regulates homeobox D10 tumor suppression pathway which can increase metastasis of cancer cells [40]. Knockdown of CCN5 in MCF-7 cells induced estrogen-independent growth, which was associated with decreased ERα expression and increased EMT [55]. In ER-negative breast cancer, glucocorticoid-induced upregulation of WISP-2 results in reduction in cellular proliferation and invasive phenotype [54].

### 6.6. CCN6: WISP-3

CCN6 plays a crucial role in tumor suppression in certain cancers, including breast cancer. WISP-3 is expressed in the normal mammary epithelium but is downregulated or absent in some invasive carcinomas [56]. One study found that its levels decreased significantly in invasive breast cancer, especially in breast cancer with axillary lymph node metastases and inflammatory breast cancer. WISP-3 is important for maintaining normal epithelial morphology in breast cancer cells. In human mammalian epithelial cells, knockdown of WISP-3 leads to resistance to anoikis and increases anchorage-specific growth. Overexpression of WISP-3 decreases invasion and distant metastasis of breast cancer cells, while its downregulation disrupts acinar morphogenesis and cell invasion in propagated mammary epithelial cells [15].

CCN6 act as a tumor suppressor in breast carcinoma [10]. Based on the study by Kleer et al., CCN6 protein has recently been shown to play an important role in the development and progression of other types of cancer [57]. CCN6 diminishes the IGF-1R signaling pathway to reduce or pause the invasion of breast cancer. IGF-1R downregulation inhibits the ZEB1 and IGF-1 expression, consequently inhibiting the E-cadherin expression [42]. As E cadherin is a cell adhesion molecule, downregulation of E cadherin can result in cancer cell metastasis. Exogenous CCN6 or small interfering RNA molecules treatment can reduce the effect of CCN6 inhibition, making it a modulator of breast cancer invasion and metastasis [58].

## 7. MicroRNA Regulation of CCN Gene in Breast Cancer

MicroRNA, commonly referred as miRNA, are small noncoding RNA molecules of ~25 nucleotides which can regulate gene expression by binding with 3′ untranslated region of mRNA [59]. MiRNA has emerged as a potential regulator of all aspects of breast cancer (see Table 2) [60].

## 8. Pharmacological Advances in Treating Breast Cancer by Targeting CCN Proteins

**Figure 2:**
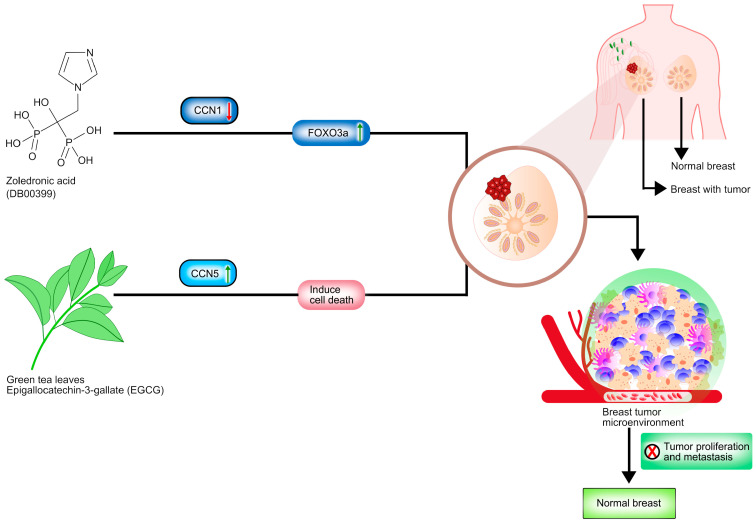
Potential pharmacological agent for breast cancer therapies. This schematic diagram illustrates Zoledronic acid (ZOL) and Epilgallocatechin-3-gallate (EGCG) obstructing breast cancer progression and metastasis by targeting CCN1 and CCN5 proteins. ZOL, DEX and EGCG play significant roles in inhibiting breast tumor formation, proliferation and metastasis. ZOL inhibits the CCN1 protein expression through FOXO3a activation in the Triple Negative Breast Cancer. DEX reestablished the CCN5 expression, which can block the TGF-β signaling pathway associated with EMT and induce metastasis suppressor protein Nm23-H1 in the ER-negative breast cancer and the EGCG from green tea, suppresses the CCN5 protein expression in the Triple Negative Breast Cancer.

Crosstalk between tumor cells and the tumor microenvironment activates a variety of critical signaling pathways, accelerating cancer progression. The CCN family proteins contain six members that may regulate the tumor microenvironment. Numerous studies have suggested CCN family proteins can be an effective target in treating breast cancer (see Figure 2) [24].

CCN1 is a well-established prognostic factor for breast cancer, especially for Triple Negative Breast Cancer (TNBC) [61]. Thus, therapeutic measures have been developed targeting CCN1. Zoledronic acid (ZOL) which is used for osteoporosis management, is now on trial as a breast cancer chemotherapeutic agent [61]. ZOL (DB00399) prevents lesion formation in bone tissues [62]. The drug has a profound impact on inhibiting breast cancer metastasis to bone. As a chemotherapeutic agent, ZOL is found to inhibit breast tumor formation, proliferation and metastasis to the bone by inhibiting CCN1 expression and FOXO3a activation [63]. Conventional dosing (intravenous administration of 4 mg drug/3–4 weeks) has been approved as it has proven in vitro and in vivo anti-tumor activity. Maintenance dosing (intravenous administration of 4 mg drugs/3–6 months) can improve the prognosis in low estrogen environments such as post-menopausal breast cancer patients. Metronomic dosing (intravenous administration of 1 mg drug/week) is suggested to have a stronger anti-tumor effect [64].

CCN5 acts as a tumor suppressor in the breast cancer cell lines. Thus, inducing CCN5 activity in breast cancer tissues can be a promising therapy against breast cancer. In vitro studies have found that epigallocatechin-3-gallate (EGCG), the polyphenol in green tea, is found to inhibit TNBC progression by activating the cell-death program [65]. Exertion of anti-tumor activity of EGCG (DB12116) is inferred to be mediated by CCN5. CCN5 might also activate ER-α and sensitize TNBC cells to hormonal therapies. EGCG promotes cell death and consequently reverses epithelial-mesenchymal transition. However, the regulation of EMT by EGCG in TNBC is quite inconsistent. Thus, a nanostructure-based drug delivery system is proposed to ensure bioavailability [65]. Conventional dosing (940 mg EGCG daily) has been approved as it has been proved through an investigation of 31 breast cancer patients conducted between 2008 and 2009 at the Los Angeles, University of Southern California Medical Center [66]. Glucocorticoid treatment of ER-negative breast cancer results in decreased invasion through the reestablishment of CCN5 expression and also induces metastasis suppressor protein Nm23-H1 [41]. Dexamethasone (DEX), a synthetic Glucocorticoid, is widely used to prevent chemotherapy induced nausea and vomiting and reduce toxicity. It has been introduced during and after chemotherapy for breast cancer patients [67]. In ER-negative breast cancer cells, DEX reduces proliferative and invasive phenotypes by reestablishing CCN5 expression, which can block the TGF-β signaling pathway concomitant with EMT. DEX treatment also induces metastasis suppressor protein Nm23-H1 [41]. Conventional dosing (100 μg/kg) has been approved during mouse experiment by Meng Pang et al. The human dose is 0.6 mg per adult which is the equivalent dose of 100 μg/kg [68].

The identification of therapeutic approaches for breast cancer treatment is a strenuous and costly process. Some drugs such as Vinblastine (DB00570), Pemetrexed (DB00642), Cladribine (DB00242), Hydroxyurea (DB01005), Docetaxel (DB01248), Trimethoprim (DB00440), Gallium nitrate (DB05260), Dactinomycin (DB00970), Eribulin mesylate (DB08871), Tenecteplase (DB00031), Nadroparin (DB08813), Regorafenib (DB08896), Pralatrexate (DB06813), etc., are related to breast cancer treatment [69], but none of these are CCN protein targeting drugs. Substantial research is needed to develop promising therapeutic options for the treatment of breast cancer targeting CCN proteins.

## 9. Future Perspectives and Limitations

Although massive research has been done on CCN protein, many questions remain unanswered regarding CCN protein functions. In our study, the approach was to bridge the research gaps on CCN protein, although previous studies have revealed paradoxical roles of different members of the CCN family.

Evidence suggests that binding of CCN1 protein with integrin αV can stimulate VEGF and TGF-β, but the pathway fo4 modulating VEGF remained unclear [35]. CCN1 protein is seen to downregulate breast cancer progression by binding with Tumor Necrosis Factor (TNF). However, the exact mechanism by which CCN1 binds to TNF remains unclear [13]. Still, the viability of other therapeutic measures targeting CCN1 protein has not been studied explicitly.

CCN2 is found to mediate TGF-β and MMP primarily. The association of CCN2 protein with integrins has been observed to prevent cell adhesion, but the establishment of the pathway via which binding of CCN2 with integrins can facilitate cellular motility is of great interest [70]. As the “complete” expression of CCN3 protein is found to exert anti-tumor activity, the detailed pathway of anti-tumor activity remains obscure [12]. CCN3 can be found in the nucleus and hypothesized to regulate gene expression, although the explicit study is required to completely understand the mechanism. A similar phenomenon has also been observed in case of CCN5 protein [71]. Despite exerting almost similar effects as CCN1, CCN2 and CCN3 have not been studied as potential therapeutic targets for breast cancer. CCN4 is one of the least explored matricellular proteins of the CCN family. The role of CCN4 in tumorigenesis and breast cancer is not only paradoxical but also ambiguous. Evidence suggests that CCN4 can induce tumorigenesis and metastasis of breast cancer [9]. On the other hand, the tumor-suppressing role of CCN4 has also been observed in other cancers [72]. Researchers need to study the tumor-suppressive effect of the CCN4 protein in the treatment of breast cancer. CCN5 protein is observed to eliminate breast cancer residues in triple negative breast cancer (TNBC) as well as exerting anti-tumor activities. More clear notion on CCN5 activity is required in order to develop therapeutic measures mediated by CCN5 proteins [65]. Among all the six CCN proteins, CCN6 is the least explored matricellular protein. CCN6 has been shown promise in inhibiting breast cancer. A significant amount of research is required in order to understand the complete mechanism of the CCN6 protein. By this means, therapeutic measures in treating breast cancer might be developed by stimulating the CCN6 effect.

Although many CCN protein functions remain unclear, some drugs have been approved to treat breast cancer metastasis and initiation by mediating CCN proteins. A clear notion of all the CCN proteins can pave the way in developing new therapeutics for treating breast cancer.

## 10. Conclusions

The role of CCN proteins is usually studied in terms of development, angiogenesis and fibrosis, but there is convincing evidence of their critical and vital role in breast tumorigenesis. Our article seeks to establish a link between CCN proteins and breast cancer. Following a review of the literature, we arrive at the following findings. The expression profiles of CCN proteins vary in the breast tissue. It is evident that numerous cellular functions are regulated by CCNs. Even though all six members of CCNs share the same protein structure, their role is strikingly different [3]. Breast cancer often develops from several sources such as an E2-dependent, non-metastatic, antiestrogen-sensitive phenotype to an E2-independent, antiestrogen-resistant, highly invasive, and metastatic phenotype [73,74]. Thus, a single member of the CCN family can be oncogenic or tumor suppressive depending on the nature of the breast tumors [8]. Furthermore, deregulated expression of CCN proteins is frequently involved in tumorigenesis and cancer progression. While the expression of CCN proteins varies in different cancer types, CCN1, CCN2, CCN3 and CCN4 participate in tumor development and act as oncogenes, but CCN1, CCN3, CCN5, and CCN6 inhibit tumor growth and play tumor-suppressive roles in breast carcinomas [2,11,12]. It can be said that CCN1 and CCN3 play a dual role in breast cancer. However, more than a decade of laboratory research has shown that CCN6 plays an inhibitory role in the growth, metastasis, and invasion in breast cancer [43,58]. The same CCN protein can perform different functions during cancer formation, maintenance, and progression. Accurately examining the expression of the whole CCN protein family at different stages of breast cancer provides a clear idea of their significance. This understanding would provide a basis for the development of new biomarkers to improve early detection and prognosis of the disease and assist in clinical assessment and intervention. Nevertheless, other matricellular proteins significantly affect various pathogenesis. Understanding the importance of ECM and its proteins may help avoid carcinogenesis and reverse inflammation. Mediators of cancer microenvironment continue to provide opportunities to explore the important clinical implications for a better fight against breast cancer [8]. Moreover, their roles in angiogenesis, tumor growth and other diseases are important areas for future research, and a complete understanding of their involvement in these processes may suggest new therapeutic strategies.

## Figures and Tables

**Table 1:** The numerous proteins and pathways implicated in breast cancer facilitated by CCN protein.

Proteins	Role	Upregulated Factors	Downregulated Factors
CCN1	Initiation	αV integrin proteins, [34]	N/A
	Proliferation	αV integrin proteins [34,35]	N/A
	Metastasis	MMP-1 [36]	PAR 1 [37]
	Tumor Inhibition	TNF [13]	N/A
CCN2	Initiation	N/A	N/A
	Proliferation	N/A	Binding with interleukin 11, BMP-9 [38]
	Metastasis	TGF-β, ERK [3]	Binding with interleukin 11, BMP-9 [38]
	Tumor inhibition	N/A	N/A
CCN3	Initiation	N/A	N/A
	Proliferation		N/A
	Metastasis	Ca oscillation, Receptor activator of nuclear factor kappa-Β ligand (RANKL)/Osteo-protegrin (OPG) [11]	N/A
	Tumor inhibition	TGF-β [39]	N/A
CCN4	Initiation	N/A	N/A
	Proliferation	N/A	N-myc downstream regulated 1 (NDRG) [9]
	Metastasis	N-cadherin, snail, β-catenin [8,9]	E-cadherin [8,9]
	Tumor inhibition	N/A	N/A
CCN5	Initiation	N/A	N/A
	Proliferation	N/A	N/A
	Metastasis	N/A	N/A
	Tumor inhibition	Homeobox D10 pathway, p27Kip1 [40]	TWIST 1, miR-10b [40], Epithelial mesenchymal transition (EMT) transcription [41]
CCN6	Initiation	N/A	N/A
	Proliferation	N/A	N/A
	Metastasis	N/A	N/A
	Tumor inhibition	N/A	E-cadherin expression, IGF-1R Signaling [42], BMP4/TAK1/p38 [43]

**Table 2:** MicroRNA involved in regulation of CCN gene expression.

Gene	MicroRNA	Role in Breast Cancer
CCN3	miR-30c [53]	“Complete” expression can inhibit breast cancer progression [12] whereas, overexpression leads to aggressive behavior of the cancer [11]
CCN2	miR-124-3p, miR-18a-5p, miR-145-5p [59]	Can cause migration and angiogenesis of breast cancer cells [7]

## Data Availability

The datasets used and/or analyzed during this study are available from the corresponding authors upon request.
